# *Bacillus amyloliquefaciens* G1: A Potential Antagonistic Bacterium against Eel-Pathogenic *Aeromonas hydrophila*

**DOI:** 10.1155/2011/824104

**Published:** 2011-07-05

**Authors:** Haipeng Cao, Shan He, Ruopeng Wei, Marek Diong, Liqun Lu

**Affiliations:** ^1^Key Laboratory of Exploration and Utilization of Aquatic Genetic Resources of Ministry of Education, Aquatic Pathogen Collection Center of Ministry of Agriculture, Shanghai Ocean University, Shanghai 201306, China; ^2^Education Department, Shanghai Normal University, Shanghai 200235, China; ^3^Technology Department, Shanxi Veterinary Drug and Feed Engineering Technology Research Center, Yuncheng 044000, China

## Abstract

Recent studies have revealed that the use of probiotics is an alternative to control marine aeromonas. However, few probiotics are available against *Aeromonas hydrophila* infections in eels. In the present study, a potential antagonistic strain G1 against the eel-pathogenic *A. hydrophila* was isolated from sediment underlying brackish water. Its extracellular products with antibacterial activities were shown to be stable under wide range of pH, temperature, and proteinase K. It was initially identified as *Bacillus amyloliquefaciens* using API identification kits and confirmed to be *B. amyloliquefaciens* strain (GenBank accession number DQ422953) by phylogenetic analysis. In addition, it was shown to be safe for mammalians, had a wide anti-*A. hydrophila* spectrum, and exhibited significant effects on inhibiting the growth of the eel-pathogenic *A. hydrophila* both *in vitro* and *in vivo*. To the best of our knowledge, this is the first report on a promising antagonistic *Bacillus amyloliquefaciens* strain from brackish water sediment against eel-pathogenic *A. hydrophila*.

## 1. Introduction

Eels are important warm water fish species cultured in several European countries including Italy, Spain, Germany, Denmark, and the Netherlands, as well as in Japan, Taiwan, Malaysia, and China [1]. Among the cultured eels, *Anguilla anguilla* (L.) is one of the most important commercial fish species, especially in the brackish Comacchio lagoons of the northern Adriatic Sea [2]. For decades, outbreaks of infectious diseases caused by *Aeromonas hydrophila* are considered to be a major economic problem to the aquaculture and quality of *Anguilla anguilla* (L.), leading to severe losses in the production and marketing of *A. anguilla* (L.) [3, 4]. At present, aeromonas can be partially controlled by fish farmers with crude application of antibiotics such as terramycin and florfenicol. However, antibiotic treatment is cost-prohibitive to farmers in many undeveloped and developing countries, and antibiotic use may be detrimental to the environment and human health, involving the development and transfer of antibiotic resistance to other aquatic bacteria, fish pathogens, human pathogens, and the accumulation of antibiotic residuals in the products [5]. Thus, besides the alternative control strategies such as improved husbandry and water quality, better nutrition, lower stocking densities, the use of beneficial microorganisms is also widely expected to become an alternative method for the prevention and control of aeromonas.

Microbial antagonism is a common phenomenon in nature [6] and plays a major role in reducing or eliminating the incidence of opportunistic pathogens in the gastrointestinal tract of aquatic animals [7]. Recently, the application of *Bacillus* sp. as a probiotic species for controlling aquatic pathogens shows promise [8]. For example, Sugita et al. isolated a *Bacillus* strain that was antagonistic to 63% of the isolates from fish intestine [9]. Sun et al. obtained two dominant gut *Bacillus* strains with antagonistic activity that could improve growth performance and immune responses of grouper *Epinephelus coioides* [10]. However, no information is available about *B. amyloliquefaciens* as a biocontrol agent for aquatic pathogens.

In this study, we isolated a *B. amyloliquefaciens* strain G1 antagonistic to the eel-pathogenic *A. hydrophila*, determined its taxonomic position, observed the physicochemical properties of its extracellular products, and assayed its *in vitro* and *in vivo* growth inhibition effects on *A. hydrophila*, and its antagonistic spectrum and pathogenicity. The data could establish its potential as an environmentally friendly probiotic for eel aquaculture.

## 2. Materials and Methods

### 2.1. Sample Collection and Isolation of Marine Bacteria

Brackish water sediment samples were collected from perch and white shrimp farms located at Qingpu District, Shanghai China. The samples were kept in a refrigerator until use. One gram of the sediment was suspended in 100 mL of autoclaved filtered brackish water, heated for 10 min at 80°C to destroy vegetative bacteria and fungi to facilitate isolation of bacilli with spores that survived the heat pretreatment. Sediment samples were then incubated in a shaker incubator (Thermo Forma Co. Ltd., USA) at 30°C with shaking at 200 rpm for 30 min. Mixtures were allowed to settle, serial dilutions up to 10^−4^ were prepared using sterile distilled water and agitated with a vortex (Hushi Laboratory Equipment Co. Ltd., Shanghai) at 200 rpm. Isolation of bacteria from this mixture was done with serial dilution technique on brackish water nutrient agar (NA) (Sinopharm Chemical Reagent Co. Ltd., Shanghai) medium. Purification of bacteria was done using repeated streaking and single colony culture at 30°C. Bacterial isolates were subcultured and transferred to brackish water NA slants. Until further use, the slants were kept at 4°C as described by Das et al. [11].

### 2.2. Screening of Antagonistic Bacteria

#### 2.2.1. Indicator Bacterium

*A. hydrophila* strain ZN1, the pathogen of septicemia in European eel *Anguilla Anguilla* (L.) [12], was obtained from Fujian Institute of Aquatic Product in Freshwater.

#### 2.2.2. Antibacterial Activity Assay

The antibacterial activities of all the bacterial isolates were examined against the eel-pathogenic *A. hydrophila* strain ZN1 by the paper disc method [13]. Briefly, a culture of *A. hydrophila* was independently spread on brackish water NA plates, then the 5 mm sterile paper discs containing the bacterial isolate with a cell density of 10^6^ cfu/disc were placed on the brackish water NA plates. Control plates consisted of *A. hydrophila* only. Zones of inhibition around the paper discs were observed and recorded on *A. hydrophila* lawn culture plates after two days of incubation at 30°C.

### 2.3. Phenotypic Characterization and Identification

The isolate was grown on brackish water NA plates (Sinopharm Chemical Reagent Co., Ltd.) at 30°C for 24 h, and then the bacterial suspension was used to inoculate the 50 CHB/E API strip (Bio-Merieux, SA) following the manufacturer's instruction. The strip was incubated at 30°C and observed after 48 h for checking against the API identification index and database.

### 2.4. Molecular Identification

#### 2.4.1. DNA Extract, PCR, and Sequencing

The Genomic DNA was extracted from a pure culture of the isolate using a genomic DNA extraction kit following instructions of the manufacturer (Shanghai Sangon Biological Engineering Technology & Services Co., Ltd.). The 16S rRNA gene fragments (ca. 1.5 kb) were amplified by PCR using a pair of universal bacterial 16S rRNA gene primers (27f): 5′-AGAGTTTGATCCTGGCTCAG-3′ and (1492r): 5′-TACGGCTACCTTGTTACGACTT-3′. The PCR was carried out according to Nduhiu et al. [14]. Briefly, 1 *μ*L of the DNA extract was amplified in a 25 *μ*L reaction mix containing 16.75 *μ*L sterilized distilled water, 2.5 *μ*L deoxyribonucleoside triphosphate (dNTP 10 mM), 2.5 *μ*L 10x buffer, 1 *μ*L MgCl_2_ (50 mM), 0.5 *μ*L of each primer (10 mM), and 0.25 *μ*L (1 U) ExTaq DNA polymerase. Amplification was done using 35 cycles of denaturation at 95°C for 1 min, annealing at 60°C for 1 min, and extension at 72°C for 1.5 min followed by a final extension 72°C for 7 min using a PCR minicycler (Eppendorf Ltd., Germany). The PCR product was electrophoresed on 1% agarose gel and visualized via ultraviolet transillumination. Sequencing was performed by a fluorescent labeled dideoxynucleotide termination method (with BigDye terminator) on ABI 3730 automated DNA Sequencer.

#### 2.4.2. Phylogenetic Analysis

The partial 16S rRNA sequence was assembled using MegAlign, Editseq, and Seqman software with a Macintosh computer. Searches were done against the National Centre for Biotechnology Information (NCBI) database using the Basic Local Alignment Search Tool (BLAST) program. The phylogenetic tree based on the 16S rRNA gene sequence of the isolate was constructed using the neighbor-joining method.

### 2.5. Physicochemical Analysis of Extracellular Products

#### 2.5.1. Preparation of the Extracellular Products

The isolate was incubated in 400 mL of brackish water nutrient broth (NB) (Sinopharm Chemical Reagent Co. Ltd., Shanghai) medium at 30°C with shaking at 200 rpm until the cell density reached 10^9^ cfu/mL. Then the cultured medium was centrifuged at 8000 rpm at 4°C for 20 min, the supernatant containing the antagonistic substance was extracted, and the extracellular products (ECPs) were obtained as described by Bordoloi et al. [15]. Briefly, the supernatant was extracted twice with equal volumes of ethyl acetate (1 : 1). The crude extract was dried over sodium sulfate and then evaporated under vacuum.

#### 2.5.2. PH Stability Assay

The influence of pH on the stability of ECPs was measured in the pH range of 5.0 to 9.0 as described by Lee et al. [16]. Briefly, the 10 mg of ECPs was added to 50 *μ*L of 50 mM citric acid buffer (pH 5), potassium phosphate buffer (pH 6–8), and carbohydrate buffer (pH 9) (Sinopharm Chemical Reagent Co. Ltd., Shanghai), then each mixture was applied to *A. hydrophila* strain ZN1 lawn cell plates. Zones of inhibition were recorded on *A. hydrophila* lawn culture plates.

#### 2.5.3. Thermal Stability Assay

The analysis on the thermal stability of ECPs was examined as described by Lee et al. [16]. Briefly, the 10 mg of ECPs was treated independently at 20, 40, 60, 80, and 100°C for 30 min. Then each treatment sample was applied to *A. hydrophila* strain ZN1 lawn cell plates. Zones of inhibition were recorded on *A. hydrophila* lawn culture plates.

#### 2.5.4. Enzyme Stability Assay

The 10 mg of ECPs was digested with 15 *μ*L of proteinase K (974 U/mL) (Shanghai Sangon Biological Engineering Technology and Services Co. Ltd.) at 30°C for 2 h. Then the processed sample was applied to *A. hydrophila* strain ZN1 lawn cell plates. Zones of inhibition were recorded on *A. hydrophila* lawn culture plates.

### 2.6. *In Vitro* Pathogen Growth Inhibition Assay

The assay was carried out in twelve 250 mL glass flask supplied with 98 mL of brackish water NB medium, and each treatment consisted of three flasks. In each flask, 1 mL of the isolate's suspension with a final cell density of 10^3^ cfu/mL, 10^4^ cfu/mL, 10^5^ cfu/mL, and 1 mL of the *A. hydrophila* suspension with a final cell density of 10^3^ cfu/mL were independently inoculated in 98 mL of brackish water NB medium, then the mixtures were incubated at 30°C with shaking at 200 rpm. The control group consisted of *A. hydrophila* strain only. Cell growth of* A. hydrophila* was measured using brackish water RS medium (Beijing Land Bridge Technology Co. Ltd.) at 24 h intervals.

### 2.7. Antagonistic Spectrum Assay

Eight pathogenic strains of *A. hydrophila* (ATCC7966, X1, S1, T3, R402L, RK1119, 706C, and 40142G) were obtained from National Collection Centre for Aquatic Pathogens, China. The antagonistic spectrum of the isolate was checked against the eight pathogenic *A. hydrophila* strains by the paper disc method [13]. The antagonistic activity against *A. hydrophila* strain ZN1 was served as the control. Zones of inhibition were observed and recorded on *A. hydrophila* lawn culture plates after two days of incubation at 30°C.

### 2.8. Virulence Assay

Hemolytic activity assay was carried out with brackish water rabbit blood agar (RBA) plates (Sinopharm Chemical Reagent Co., Ltd.) at 30°C for 2 days. Virulence was further assayed in mice. Briefly, four-week-old female BALB/c mice, weighing 20 g each, were obtained from Laboratory Animal Centre of Second Military Medical University, Shanghai. Mice were lightly anesthetized with Halothane (Sinopharm Chemical Reagent Co., Ltd.) in a glass desiccator and challenged with the isolate's suspension prepared as mentioned above. The isolate's suspension was orally administered at the final cell density of 10^5^, 10^6^, 10^7^, 10^8^, and 10^9^ cfu/g through a micropipette fitted with a fine micropipette tip and thin flexible tube. The control mice were orally administered with the autoclaved brackish water NB medium. Ten mice were tested in each dilution. The mice were housed in cages at 20–25°C, fed with the pellet feed and purified water. Mice were examined daily and any signs of disease and mortality were recorded up to 14 days. The 50% lethal dose (LD_50_) was determined according to Mittal et al. [17].

### 2.9. *In Vivo* Protection Test

Ninety *Anguilla anguilla* (L.), weighing 90–100 g each, were allowed to acclimatize for 7 days and were randomly placed in three 200 L tanks (10 fish per tank, three tank per group) for the three treatments (the control, low cell density, and high cell density groups) described below. The tanks used recycled brackish water that was kept at 28°C throughout the experiment. The isolate's suspension was prepared as mentioned above and its cell densities were determined. Under sterile conditions, the isolate was manually incorporated into commercial dry pellets at rates of 3 × 10^7^ and 3 × 10^9^ cfu/g in feed for low and high cell densities of the isolate diets, respectively. Fish fed only commercial dry pellets served as a control. Fish were fed approximately 1% of body weight once a day. Two weeks after feeding, all the fish were bath-challenged with skin scarification through exposure to *A. hydrophila* strain ZN1 with a final cell density of 10^9^ cfu/mL as recommended by Schadich and Cole [18]. Briefly, all the fish were skin scarified, the skin scarified fish in the low cell density and high cell density groups were exposed to the suspension of *A. hydrophila* strain ZN1 overnight, while the skin scarified fish were exposed individually to brackish water only. After the bacterial exposure, the fish were returned to their living containers. Dead fish were immediately removed for pathogen isolation as described by Bucke [19], and mortalities were recorded each day for 14 days following the immersion challenge.

### 2.10. Statistical Analysis

Data were presented as the mean ± standard deviation (SD) for the indicated number of independently performed experiments. *P* < 0.05 was considered statistically significant using one-way analysis of variance.

## 3. Results

### 3.1. Isolation of Marine Antagonistic Strains

A total of 45 bacteria were isolated from the brackish water sediment samples. Only one isolate, named G1, was found to exhibit strong antagonistic activity to the eel-pathogenic *A. hydrophila* strain ZN1, displaying inhibition zones of 15 mm (data not shown). According to Lategan et al. [20], zones of inhibition >12 mm against *A. hydrophila *were considered as susceptibility to the isolates. Thus, isolate G1 was chosen for further study.

### 3.2. Characterization and Identification

The API identification kits identified isolate G1 as *Bacillus amyloliquefaciens* (data not shown), and it showed an identity of 94% with the type strain ATCC23350 in phenotypic characterization. Isolate G1 and the type strain ATCC23350 were found both positive for glycerin, L-Arabinose, D-Ribose, D-Xylose, D-Galactose, D-Glukose, D-Fructose, D-Mannose, inositol, D-Mannitol, D-Sorbitol, Methyl-*α*D-Glucopyranoside, amygdalin, arbutin, esculin, salicin, D-Cellobiose, D-Maltose, D-Lactose, D-Melibiose, D-Saccharose, D-Trehalose, D-Melezitose, D-Raffinose, amidon, glycogen, and gentiobiose. However, there were some differences between isolate G1 and the type strain ATCC23350. For example, in contrast to the type strain ATCC23350, isolate G1 was unable to ferment inulin.

**Figure 1:**
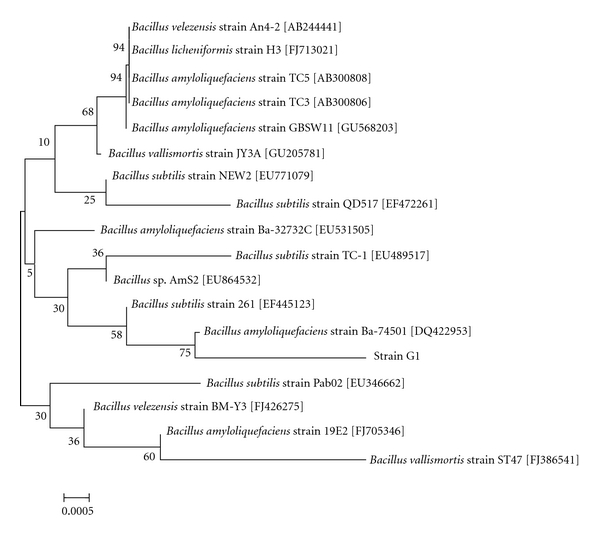
Phylogenetic tree constructed using neighbor-joining method.

The 1.5 kb 16S rRNA sequence of isolate G1 was submitted to GenBank database with the accession number HM245965. Similarities between the 16S rRNA sequence of isolate G1 and those of *B. amyloliquefaciens* strains in the GenBank database were 99.0%, which proved the initial identification. The constructed phylogenetic tree using neighbor-joining method further demonstrated that isolate G1 was closely related to the *B. amyloliquefaciens* strain (GenBank accession number DQ422953) (Figure 1). The identification result from phylogenetic analysis was consistent with that found through API identification kits.

### 3.3. Physicochemical Properties of the Extracellular Products

The ECPs were obtained from the supernatant (pH 8.5) of isolate G1 with ethyl acetate. The ECPs could inhibit the growth of the eel-pathogenic *A. hydrophila*, creating the clear inhibition zone on the *A. hydrophila* lawn culture plate (data not shown). The antagonistic activity of the ECPs was retained over the wide pH range of 5.0 to 9.0 against *A. hydrophila* strain ZN1, and it was also thermally stable at up to 100°C for 30 min, both showing no significant difference between the inhibition zones (data not shown). In addition, the antagonistic activity of the ECPs was still retained with proteinase K treatment, forming a clear zone on the *A. hydrophila* lawn culture plate (data not shown). The results indicated that the antagonistic substance in the ECPs was stable under wide range of pH, temperature, and proteinase K.

### 3.4. *In Vitro* Growth Inhibition Effect

**Figure 2:**
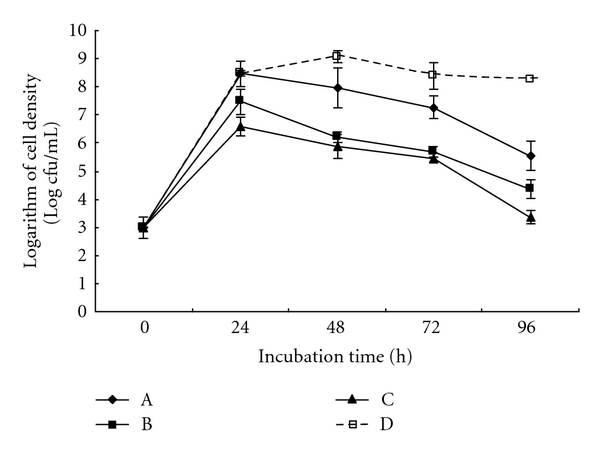
Inhibitory effect of srain G1 at the final cell density of 10^3^ cfu/mL (A), 10^4^ cfu/mL (B), 10^5^ cfu/mL (C), and 0 cfu/mL (D) on the growth of the eel-pathogenic *A. hydrophila*.

The *in vitro* effect of isolate G1 on the growth inhibition of *A. hydrophila* strain ZN1 was shown in Figure 2. The cell density of *A. hydrophila* was significantly lower than that in the control when isolate G1 was inoculated at the final cell density of 10^3^ to 10^5^ cfu/mL, and the logarithms of the cell density of *A. hydrophila* were, respectively, reduced by 32.65%, 47.28%, and 59.49% after the incubation of 96 h, compared with the control group. The result indicated that isolate G1 could be used for exclusion of *A. hydrophila.*

### 3.5. Antagonistic Activity against *Aeromonas hydrophila* Strains

**Figure 3:**
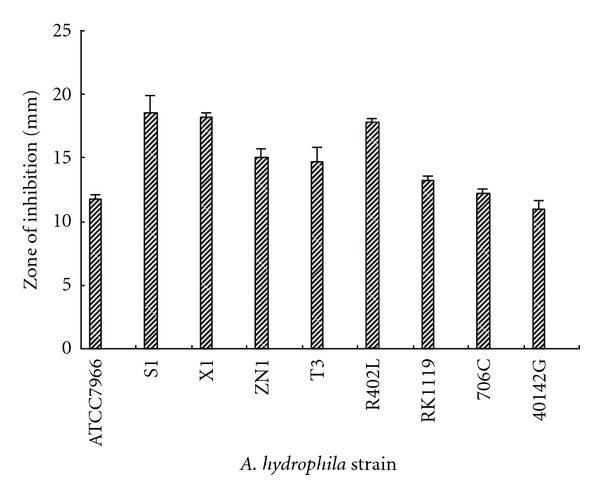
Antagonistic spectrum of srain G1 against pathogenic* A. hydrophila* strains.

The antagonistic activity against the eight pathogenic *A. hydrophila* strains was shown in Figure 3. The result indicated that isolate G1 had highly antagonistic activity against the other pathogenic *A. hydrophila* strains besides *A. hydrophila *strain ZN1. The maximum zone of inhibition (18.5 mm) was recorded in strain S1, and followed by strain X1 (18.25 mm), strain R402L (17.75 mm), strain ZN1 (15 mm), strain T3 (14.75 mm), strain RK1119 (13.25 mm), and strain 706C (12.25 mm). According to Lategan et al. [20], zones of inhibition >12 mm against *A. hydrophila *were considered as susceptibility to the isolate. Therefore, isolate G1 had a wide antagonistic spectrum against pathogenic *A. hydrophila* strains. 

### 3.6. Safety

No hemolytic activity was detected with isolate G1, with no zones of hemolysis being formed on the RBA plates (data not shown). In addition, no acute mortality or any visible disease signs were observed in the test mice treated with 10^5^ to 10^9^ cfu/g of isolate G1's suspension (data not shown). It is concluded that the LD_50_ value of isolate G1 was estimated to exceed 10^9^ cfu/g according to Mittal et al. [17].

### 3.7. *In Vivo* Protective Effect

**Figure 4:**
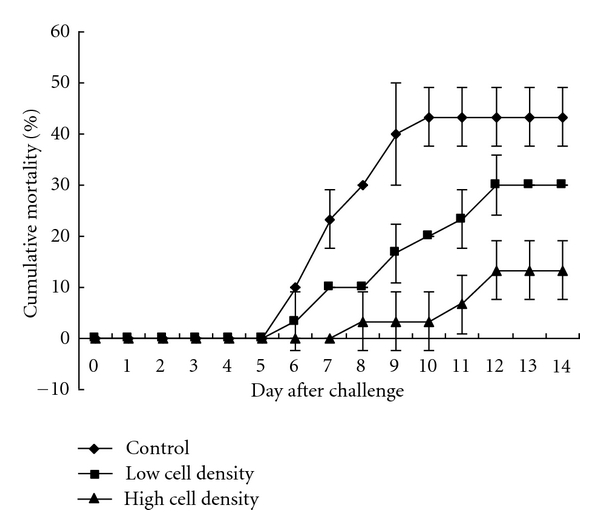
Protective effect of srain G1 on *Anguilla anguilla* (L.) under the eel-pathogenic *A. hydrophila* challenge trial.

The *in vivo* protective effect of isolate G1 on *Anguilla anguilla* (L.) under the eel-pathogenic *A. hydrophila* challenge trial was shown in Figure 4. After 14 days following the immersion challenge, the cumulative mortality was 69.24% lower in the high cell density group than in the control group, and the cumulative mortality was also 30.76% lower in the low cell density group than in the control group. The death of all the test fish observed in the challenge trials was caused by *A. hydrophila*, as determined by bacterial isolation and API identification kits (data not shown). The result indicated the protective effect of isolate G1 against *A. hydrophila *infection in *Anguilla anguilla* (L.).

## 4. Discussion

The use of antagonistic bacteria is widely expected to become an alternative method for the prevention and control of bacterial disease in fish. Numerous studies have shown that bacteria can produce inhibitory substances that had the effect of inhibiting the bacterial pathogens in aquaculture systems [13]. The use of such bacteria to inhibit pathogens by release of antimicrobial substances is now gaining importance in the eel farming as a better and more effective alternative than administering antibiotics to manage the health of eels [18]. The present study reported a promising antagonistic *B. amyloliquefaciens* isolate G1 from the brackish water sediment samples, which showed antagonistic property towards the eel-pathogenic *A. hydrophila* and other pathogenic *A. hydrophila* strains. Our data indicated that the isolate could be a suitable candidate probiotic for eel farming: (1) a significant *in vitro* inhibitory effect on the growth of eel-pathogenic *A. hydrophila*; (2) a significant *in vivo* protective effect against *A. hydrophila* infection in *Anguilla anguilla* (L.); (3) stability of the antagonistic action of its extracellular products over a wide range of pH, temperatures, and proteinase K. 

In the present study, the extracellular products (ECPs) of isolate G1 showed inhibitory activity on the eel-pathogenic* A. hydrophila* strain ZN1 (data not shown), and the inhibitory activity of the ECPs was not significantly affected under wide range of pH, temperature, and proteinase K. Relevant studies indicated that the antagonistic action responsible for the inhibition of bacterial pathogens such as *Erwinia amylovora*, *Ralstonia solanacearum* was due to difficidin, bacilysin, or a 29 kDa fusion protein of the *LCI* gene [21, 22]. The production of antibiotic substance by isolate G1 might be one of these important inhibiting agents. To clarify this, further characterization of the inhibitory component of isolate G1 would be necessary. In addition, the inhibitory activity of the ECPs of isolate G1 even after treatment at high temperatures (data not shown) and proteinase K (data not shown) suggested the stability of the antagonistic component. Similar observations were also recorded by Hu et al. [22], who reported that the antibacterial activity of the active fractions of *B. amyloliquefaciens* isolate Bg-C31 was not affected at 100°C and proteinase K. The property of thermal stability would be useful during industrial level production.

For application of isolate G1 as a probiotic during routine hatchery operations and natural *A. hydrophila* infections, the data on the effect of inhibiting the growth of *A. hydrophila* were essential. The present study indicated that isolate G1 could significantly reduce the cell density of *A. hydrophila* by 32.65%, 47.28%, and 59.49% after the inoculation at the final cell density of 10^3^ to 10^5^ cfu/mL, respectively (Figure 2), and produced a maximum inhibition zone with 18.5 mm on *A. hydrophila* lawn culture plates (Figure 3). In a related study on the antibacterial activity of *Bacillus *sp., *Bacillus subtilis* strain P73 and strain P72 only exhibited a maximum inhibition zone with 14.5 mm on *A. hydrophila* lawn culture plates [23]. Therefore, isolate G1 might be considered as a stronger antagonistic bacterium. 

In order to be considered as a probiotic for application, the candidate strain had to be evaluated for its pathogenicity to a mammalian system and protective effect [24]. The present study showed that isolate G1 could not form any hemolytic rings on the RBA plates (data not shown), and the LD_50_ value to BALB/c mice exceeded 10^9^ cfu/g. As described by Cutting [25], the *Bacillus* strain was regarded as no infectivity or toxicity when its oral LD_50_ value to mice is above 4.7 × 10^8^ cfu/g. Thus, isolate G1 was evaluated as a safe strain. Supplementation of *Saccharomyces cerevisiae* has been used to control *Aeromonas hydrophila* infection in *Oreochromis niloticus* (L.) [26], but no relevant data are available about antagonistic bacteria against *A. hydrophila* infection in eels. The present study indicated that supplementation of isolate G1 could significantly reduce the cumulative mortality of *Anguilla anguilla* (L.) challenged with *A. hydrophila* (Figure 4), confirming the protective effect of isolate G1 against *A. hydrophila* infection in eels.

In conclusion, the unique characteristics of *B. amyloliquefaciens *G1, such as the antibacterial property towards a wide spectrum of pathogenic *A. hydrophila* strains, the significant growth inhibition effect on the eel-pathogenic *A. hydrophila*, the protective effect against *A. hydrophila *infection in *Anguilla anguilla* (L.), resistance of its extracellular products to a wide range of pH, temperatures, and proteinase K, and its safety to the mammalian system, supported this strain as a promising probiotic for the biocontrol of *A. hydrophila *infections in *A. anguilla* (L.).
